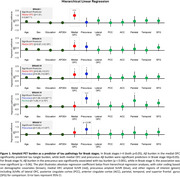# Tau burden across Braak stages is associated with regionally specific amyloid accumulation: insights from the BIOCARD cohort

**DOI:** 10.1002/alz70862_110259

**Published:** 2025-12-23

**Authors:** Nisha Rani, Kylie H. Alm, Daniel D. Callow, Corinne Pettigrew, Anja Soldan, Michael Miller, Marilyn S. S. Albert, Arnold Bakker

**Affiliations:** ^1^ Johns Hopkins University School of Medicine, Baltimore, MD USA; ^2^ Department of Neurology, Johns Hopkins University School of Medicine, Baltimore, MD USA; ^3^ Johns Hopkins University, Baltimore, MD USA

## Abstract

**Background:**

Alzheimer’s disease is characterized by accumulation of amyloid‐β (Aβ) plaques and tau tangles. Aβ deposition typically begins in neocortical regions years before onset of cognitive decline, while tau pathology spreads systematically through select brain regions, typically described as Braak stages (I‐VI). The impact of regional Aβ on tau propagation remains a key research focus.

**Method:**

We examined the associations between regional Aβ and tau burden measured by positron emission tomography (PET) in 192 participants without dementia (14 with mild cognitive impairment [MCI]) in the BIOCARD cohort, of whom 52 (27%) were Aβ positive. Amyloid status was assessed using standardized uptake value ratios (SUVR) obtained from the medial orbitofrontal cortex (OFC), lateral OFC, precuneus, posterior cingulate, anterior cingulate, parietal, temporal, and superior frontal regions using 11C‐PIB PET. Tau burden was quantified using 18F‐MK6240 PET across 29 brain regions, assigned to each of the Braak stages. Associations were examined with stepwise and hierarchical regressions, covarying for age, sex, education, and ApoE4 status.

**Result:**

In stepwise regression analyses for Braak stages (I‐II), amyloid in the medial OFC emerged as significantly associated with tau burden *(β* = 1.45, *t* = 4.75*, p* <.001 for Braak‐I; *β* = 0.80*, t* = 3.58*, p* <.001 for Braak‐II), while in later Braak stages, amyloid in the precuneus emerged as significantly associated with tau burden (*β* = 0.57, *t* = 9.59*, p* <.001for Braak‐III*; β* = 1.54*, t* = 3.76, *p* <.001 for Braak‐IV*; β* = 0.39*, t* = 5.90*, p* <.001 for Braak‐V*;* and *β* = 0.17*, t* = 2.30, *p* <.05 for Braak‐VI). Subsequent hierarchical regressions showed that including other Aβ regions did not significantly alter the proportion of variance explained for any Braak stage (Figure 1).

**Conclusion:**

Amyloid‐tau interactions show regional specificity with medial OFC amyloid burden showing a strong association with early tau pathology accumulation while amyloid in the precuneus is selectively associated with tau in later stages. These findings suggest that localized measures of amyloid burden may enhance early detection and show utility in understanding amyloid‐tau dynamics.